# Effects of Hot Air, Vacuum, and Conductive Drying on the Fatty Acid Profile of *Cucurbita maxima* Pulp and Its Processing By-Products

**DOI:** 10.3390/foods14010057

**Published:** 2024-12-28

**Authors:** Antonela Ninčević Grassino, Sven Karlović, Filip Dujmić, Suzana Rimac Brnčić, Marija Badanjak Sabolović, Mladen Brnčić

**Affiliations:** Faculty of Food Technology and Biotechnology, University of Zagreb, Pierottijeva 6, 10 000 Zagreb, Croatia; skarlovi@pbf.hr (S.K.); filip.dujmic@pbf.unizg.hr (F.D.); srimac@pbf.hr (S.R.B.); mbadanjak@pbf.hr (M.B.S.)

**Keywords:** fresh and dried pumpkin pulp, hot air drying, vacuum drying, conductive drying, pumpkin by-products, fatty acids, nutritional index

## Abstract

Considering the short shelf life of fresh pumpkin due to its high water content and the extensive use of dried pumpkin in the food industry, it is necessary to find an efficient drying method that minimizes water activity and preserves nutritional properties. In this study, the effects of hot air drying (HAD), vacuum drying (VAD), and conductive drying (CD) at 50, 60, and 70 °C on fatty acid profiles were investigated to determine optimal drying conditions that preserve fatty acid (FA) quality and associated nutritional benefits. Results showed that drying methods had a significant effect (*p* < 0.05) on fatty acid composition and yield, resulting in different amounts of palmitic, oleic, linoleic, and linolenic acids as major FAs compared to fresh pulp. The saturated FA content was higher in CD pulp (up to 42.37%), followed by HAD and VAD. Oleic acid, as the most important representative of monounsaturated FAs, came from VAD (up to 30.64%). Linoleic and linolenic acid, as the most important polyunsaturated FAs of the omega-6 and omega-3 fatty acids, were found in higher proportions in CD pulp at 50 and 60 °C (up to 31.12%) and HAD pulp at 60 and 70 °C with an airflow velocity of 1.5 m/s (up to 39.70%). In addition, the peel and seeds, the by-products resulting from the processing of the fruit pulp, were also evaluated with regard to the fatty acid profile. Two fractions also contained the four major FAs in representative amounts, indicating their valuable reuse.

## 1. Introduction

It is known that lipids are the elementary nutrients for humans and high energy reserves [1]. Lipids consist of fatty acids (FAs), which are classified according to the presence or absence of double bonds, i.e., saturated (SFAs, without double bonds), monounsaturated (MUFAs, with one double bond), and polyunsaturated (PUFAs, with two or more double bonds), and further as cis or trans fatty acids, based on the configuration of the double bonds, and as n-3 or n-6 PUFAs, depending on the position of the first double bond from the fatty acid methyl end [2,3]. The human body cannot synthesize PUFAs with the first double bond at C3 and C6 from the methyl end because it lacks the corresponding enzymes. These acids are essential for humans and must therefore be ingested with food [4].

Nowadays, nutritionists recommend vegetable oils as an important part of a healthy diet instead of animal or fish oils as traditional sources of fatty acids [5,6]. The distribution of fatty acids is variable and depends largely on the plants and their components as well as the technology used for plant processing [7,8,9]. Among the various plants, the pumpkin family (*Cucurbitaceae*) has attracted increasing attention as part of the Mediterranean diet due to its excellent nutritional profile. For example, the pumpkin species, i.e., *Cucurbita maxima*, *moschata*, and *pepo*, contain a wealth of nutrients, e.g., proteins, carbohydrates, lipids, fiber, etc., in addition to phytochemicals, e.g., carotenoids, tocopherols, fatty acids, etc., which make them valuable functional foods [10,11,12,13]. Their phytonutrient potential, which includes antioxidant, anti-inflammatory, antibacterial, anticarcinogenic, antihypertensive, and antidiabetic effects [14,15], varies and depends on the pumpkin components (pulp, peel, and seeds). For example, pumpkin seed oils, which are a product of seed processing, are good natural sources of essential fatty acids and phytosterols [16,17,18], so a diet high in monounsaturated and polyunsaturated fatty acids results in a lower risk of various heart diseases. In view of this, the content of high-density lipoprotein (HDL) cholesterol (“good” cholesterol) increases, and the low-density lipoprotein (LDL) cholesterol (“bad” cholesterol) decreases due to the high content of MUFA and PUFA. Although much attention has been paid to pumpkin seed oils as a source of FAs, their content in the raw and especially in the dried pulp has not been reported. Therefore, considering the widespread production and consumption of pumpkin and the increasing use of the pulp in the daily diet, further studies are needed to confirm the FA profile. Pumpkin can be used raw or converted into a variety of nutritious and tasty ‘convenience’ products, such as jams, juices, sauces, pickles, etc. Pumpkin pulp has a naturally sweet taste and attractive yellow-orange color, so adding dried pumpkin pulp to various dishes, e.g., breakfast cereals, snack bars, soups, instant meal smoothie powders, pasta, bread, and cookies, can improve the appearance and nutritional value of various foods [12,19].

Drying is the oldest technique for food preservation [20]. Considering the short shelf life of fresh pumpkin due to its high water content and the extended application of dried pumpkin in the food industries, e.g., meat, bakery, beverage, etc. [21,22], it is necessary to find an effective drying method [23,24,25] that reduces the activity of the water and preserves the nutritional and phytochemical profile of the pumpkin. As previously reported by Ninčević Grassino et al. [26], convective hot air drying (HAD), vacuum drying (VAD), and conductive dying (CD) have shown that it is possible to extend the shelf life of fresh pumpkin while maintaining good nutritional and texture quality of the dried pulp. In HAD drying, a closed atmosphere with controlled airflow velocity and temperature makes this method quite effective and simple. However, the extended drying time and presence of oxygen in the air lead to the degradation of valuable components that are highly sensitive to oxidation, particularly at elevated temperatures. Despite these disadvantages, HAD is still one of the most commonly used drying techniques in the food industry. Although CD ensures uniform heat transfer, which improves drying efficiency and consistency, this process, similar to HAD, can thermally damage sensitive nutrients, so it requires careful temperature management [24,26]. To avoid deterioration due to hot air drying and conductive drying, VAD under reduced pressure and lower temperatures can help preserve the heat- and oxygen-sensitive ingredients, colors, and flavors. Radojčin et al. [25], Calín-Sánchez et al. [27], and Nainggolan et al. [28] describe the main features, including mechanisms, advantages, and disadvantages, of HAD and VAD application.

Since, to our knowledge, there are no reports on the fatty acid profile in fresh and especially in dried pumpkin pulp, this study investigated the composition and content of fatty acids in fresh as well as HAD-, VAD-, and CD-processed samples. Thus, the main objective of this work was to investigate and compare three different drying methods and to select optimal parameters for obtaining dried pumpkin pulp with high-quality fatty acids for the fortification of various foods. In addition to this main objective, the present work also highlighted the FA composition of the seeds and peel, which are produced in large quantities during the preparation of the pulp for drying processes. These materials, which are usually considered as waste, can be effectively converted into valuable functional and nutraceutical components [12,29,30,31], which increases the added value of food processing and also significantly reduces environmental pollution.

## 2. Materials and Methods

### 2.1. Chemicals and Reagents

All reagents, standards, and solvents were of analytical grade. Thirty-seven component standard FAME in methylene chloride containing C4 to C24 FAME was purchased from Sigma-Aldrich (St. Louis, MO, USA). Hexane, methanol, sodium hydroxide, and sodium hydrate sulphate tetrahydrate were purchased from Kefo (Zagreb, Croatia).

### 2.2. Drying Process

Fresh pumpkin pulp of *Cucurbita maxima* was dried using different processing parameters employed in hot air drying (HAD), vacuum drying (VAD), and conductive drying (CD). The preparation of the samples for HAD, VAD, and CD was described previously by Ninčević Grassino et al. [26] and Karlović et al. [32], whereas the drying conditions were reported by Ninčević Grassino et al. [21]. Briefly, HAD was carried out at three different temperatures of 50, 60, and 70 °C and airflow velocities of 0.5, 1.0, and 1.5 m/s using an ARMFIELD UOP8-MKII dryer (Armfield Limited, Ringwood BH24 2PB, UK). VAD was performed at temperatures of 50, 60, and 70 °C and a vacuum pressure of 100 mbar using a Memmert VO 200 vacuum drying oven with a Memmert PM200 pump module (MEMMERT GmbH+Co.KG, Schwabach, Germany), and CD at atmospheric pressure of 1000 mbar at temperatures of 50, 60, and 70 °C using a Memmert VO 200 drying oven without Memmert PM200 pump module (MEMMERT GmbH+Co.KG, Schwabach, Germany). All experiments were carried out at a humidity of 55 ± 1%.

### 2.3. Determination of Fatty Acids

The total fat content was determined gravimetrically after the samples were extracted with petroleum ether for 8 h in a Soxhlet apparatus [33]. The values obtained for the fresh and dried samples have already been reported by Ninčević Grassino et al. [26], while for the peel and seeds, the total fats were 3.24 and 21.44%, respectively. Subsequently, the extracts were used for fatty acid analyses in fresh and HAD, VAD, and CD pumpkin pulp, as well as in the by-product peel and seeds.

Fatty acid methyl esters (FAME) were prepared by trans-esterification of each extract with methanolic solution of sodium hydroxide following the procedure described by ISO 15884 [34]. Briefly, 100 mg of fat was weighted into a glass tube, followed by addition of 5 mL of n-hexane. After dissolution of fat, the 0.2 mL of sodium metoxide solution (2 mol/L) was added with a micropipette. The tube contents were shaken vigorously for 1 min, and then 0.5 g of sodium hydrate sulphate tetrahydrate was added, and the tube contents were centrifuged at room temperature for 3 min. After equilibration and separation of the layers, the upper layer containing FAME was carefully removed by pipette and used for GC-FID analysis according to ISO 15885 [35].

The GC analysis was performed by gas chromatograph (Shimadzu, Tokyo, Japan) equipped with the flame ionization detector. FAMEs were separated on a 30 m × 0.25 mm, 0.25 μm film thickness, InertCap PureWax capillary column (Shimadzu, Japan). The column temperature was initially held at 50 °C (5 min), rising to 260 °C (30 min). Helium was used as a carrier gas. Injector temperature was maintained at 250 °C, and the injection volume was 1 μL with split ratio 1:50. Automatic chromatographic software solution GC, Version 2.41.00 (Shimadzu, Japan), was used for data collection and calculation by comparing retention times of standards. All fatty acid content results were reported on a dry basis.

### 2.4. Fatty Acid Nutritional Quality Index Evaluation

To evaluate the nutritional value of the fatty acids, the PUFA/SFA index, the index of atherogenicity (IA), the index of thrombogenicity (IT), and the index of hypocholesterolemia/hypercholesterolemia (HH) were calculated according to the formulas of Chen et al. [4]. In addition, the MUFA/SFA and total omega-6/total omega-3 indices were calculated according to the formula presented in the work of Montesano et al. [36].

### 2.5. Statistical Analysis

Results were expressed as mean ± standard deviation of triplicate measurements (*n* = 3). Significant differences (*p* < 0.05) within means were examined using analysis of variance (ANOVA) and Tukey’s test for honestly significant differences (HSD). All tests for data analysis were evaluated using SPSS Statistics software version 26 (IBM, New York, NY, USA).

## 3. Results and Discussion

In this work, the effects of three different drying methods (HAD, VAD, and CD) and drying parameters were investigated to find out which of them are able to preserve the fatty acid composition and provide the highest amounts in dried pumpkin pulp samples.

In this context, the fatty acid profile was analyzed and compared with the values of the fresh samples to highlight the efficiency of drying. Since there are no studies on the fatty acid composition of the dried pumpkin pulp, the results obtained in this work were compared with the results of other pumpkin fractions [10,17,18,36,37,38,39,40,41,42,43,44,45,46,47,48,49,50,51,52,53,54], mainly the seeds (Table 1).

In addition to the data for the pumpkin pulp, the fatty acid profiles in the pumpkin by-products were also evaluated, and the nutritional indices of all analyzed samples were described. They can help to show the fatty acid composition and yield, as well as their influence on the health protection value from a nutritional point of view, which would be of particular importance if pumpkin pulp is added in dried form to produce various pumpkin-based products.

### 3.1. Fatty Acid Profile of Fresh Pumpkin Pulp

**Figure 1:**
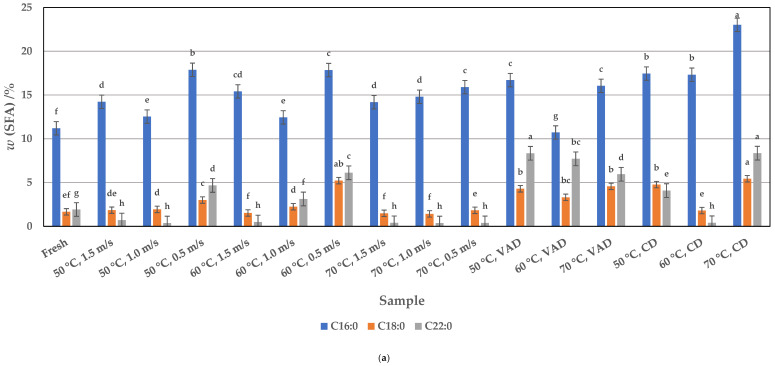

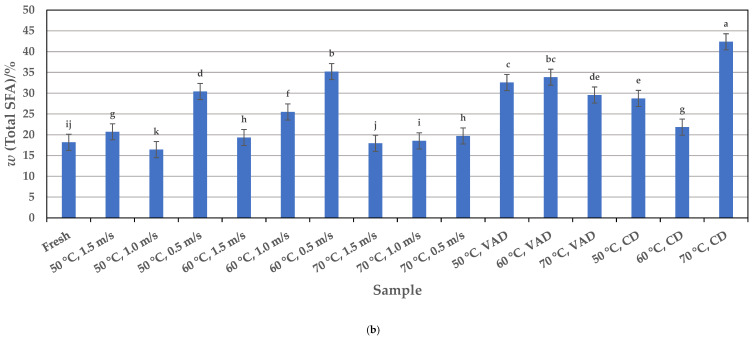
(**a**) Profile of saturated fatty acids (SFAs) in fresh and HAD, VAD, and CD pumpkin pulp. Values are means ± standard deviations of three (*n* = 3) measurements. Different lowercase letters in the same column indicate significant differences (*p* < 0.05) between drying methods (HAD—hot air drying: 50, 60, and 70 °C, and 0.5, 1.0, and 1.5 m/s, VAD—vacuum drying, CD—conductive drying under atmospheric pressure) and conditions. (**b**) Total content of saturated fatty acids (SFAs) in fresh and HAD, VAD, and CD pumpkin pulp. Values are means ± standard deviations of three (*n* = 3) measurements. Different lowercase letters in the same column indicate significant differences (*p* < 0.05) between drying methods (HAD—hot air drying: 50, 60, and 70 °C, and 0.5, 1.0, and 1.5 m/s, VAD—vacuum drying, CD—conductive drying under atmospheric pressure) and conditions.

**Figure 2:**
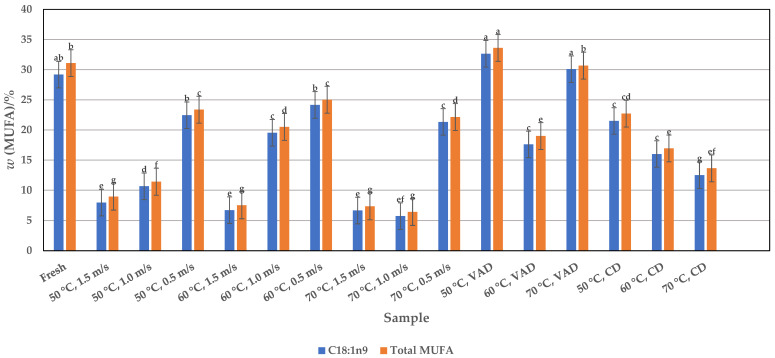
Profile of monounsaturated fatty acids (MUFAs) in fresh and HAD, VAD, and CD pumpkin pulp. Values are means ± standard deviations of three (*n* = 3) measurements. Different lowercase letters in the same column indicate significant differences (*p* < 0.05) between drying methods (HAD—hot air drying: 50, 60, and 70 °C, and 0.5, 1.0, and 1.5 m/s, VAD—vacuum drying, CD—conductive drying under atmospheric pressure) and conditions.

**Figure 3:**
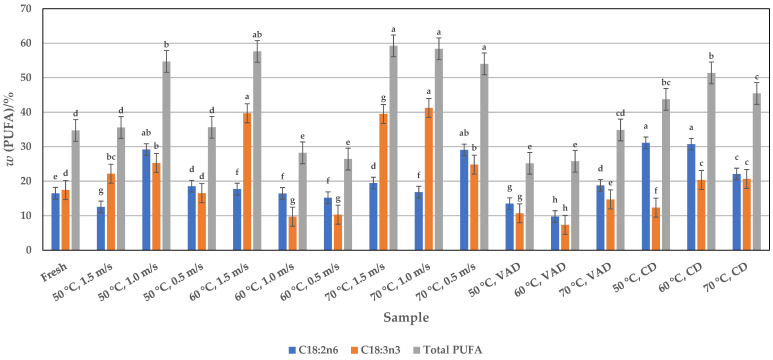
Profile of polyunsaturated fatty acids (PUFAs) in fresh and HAD, VAD, and CD pumpkin pulp. Values are means ± standard deviations of three (*n* = 3) measurements. Different lowercase letters in the same column indicate significant differences (*p* < 0.05) between drying methods (HAD—hot air drying: 50, 60, and 70 °C, and 0.5, 1.0, and 1.5 m/s, VAD—vacuum drying, CD—conductive drying under atmospheric pressure) and conditions.

The results of GC-FID analyses showed that fresh pumpkin pulp contains 18.18%, 31.08%, and 34.71% of total SFAs, MUFAs, and PUFAs, respectively (Figure 1, Figure 2 and Figure 3). The most abundant SFA in fresh pumpkin pulp is palmitic acid (C16:0), with a value of 11.20%. With this palmitic acid content, fresh pumpkin pulp can be considered a lower-fat food source compared to various other foods in which the palmitic acid content in animal fats is 20–30% and in vegetable oils, where it is 10–45% [55]. Although palmitic acid is one of the most important SFAs, the others, e.g., tetracosanoic acid (C24:0), stearic acid (C18:0), and behenic acid (C22:0), also contributed to the total SFA content, with values of 1.00%, 1.66%, and 1.92%, respectively. The influence of C6:0, C8:0, C10:0, C12:0, C13:0, C14:0, C15:0, C17:0, C20:0, C21:0, and C23:0 is negligible due to the lowest values found, i.e., 0.03 to 0.97%. Among MUFAs, oleic acid (C18:1n9) predominates with a content of 29.19%, while other MUFAs, such as palmitoleic acid (C16:1n-7), heptadecenoic acid (C17:1n7), eicosenoic acid (C20:1n9), erucic acid (C22:1n9), and nervonic acid (C24:1n9), were found in amounts of up to 0.68%. Among the PUFAs, linoleic acid (C18:2n6) and linolenic acid (C18:3n3) are the most important, as they are necessary for normal growth and development and for the physiological function of body systems [56]. Fresh pumpkin pulp contains 16.50 and 17.43% linoleic and linolenic acids, respectively, while the other essential PUFAs, such as docosahexaenoic acid (22:6n3) and eicosapentaenoic acid (20:5n3), are absent or present only in trace amounts (0.19%). These results are in line with other studies that emphasize pumpkin as a nutrient-rich crop with a high content of polyunsaturated and monounsaturated fatty acids [10,17,18,36,37,38,39,40,41,42,43,44,45,46,47,48,49,50,51,52,53,54]. Compared to other pumpkin fractions such as the seeds and peel, the fresh pulp contains a higher proportion of linolenic acid. These differences emphasize the variability in composition between the different parts of the *Cucurbita* species [10,17,18,36,37,38,39,40,41,42,43,44,45,46,47,48,49,50,51,52,53,54].

**Figure 4:**
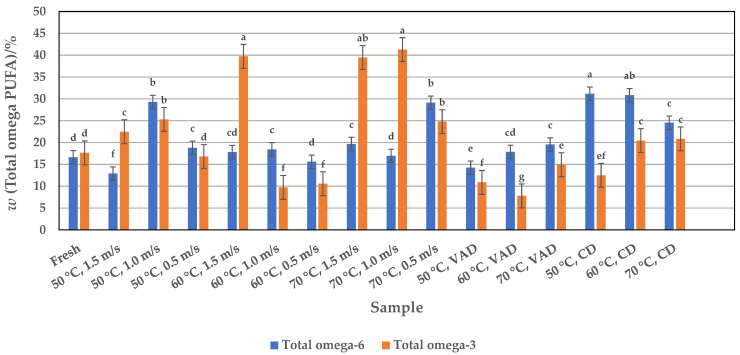
Profile of total omega-6 and total omega-3 polyunsaturated fatty acids (PUFAs) in fresh and HAD, VAD, and CD pumpkin pulp. Values are means ± standard deviations of three (*n* = 3) measurements. Different lowercase letters in the same column indicate significant differences (*p* < 0.05) between drying methods (HAD—hot air drying: 50, 60, and 70 °C, and 0.5, 1.0, and 1.5 m/s, VAD—vacuum drying, CD—conductive drying under atmospheric pressure) and conditions.

Nowadays it is generally agreed that people should consume more omega-3 and fewer omega-6 fatty acids for good health. It is not clearly known what ratio of omega-6 to omega-3 fatty acids is desirable in the diet or to what extent a high intake of omega-6 fatty acids interferes with the benefits of consuming omega-3 fatty acids [55,57]. Based on the higher proportion of total omega-3 fatty acids (17.66%) than total omega-6 fatty acids (16.64%) in fresh pumpkin pulp (Figure 4) with a ratio of 0.94, it can be hypothesized that this food source may have beneficial effects on human health. Excessive amounts of omega-6 fatty acids and a very high ratio of omega-6 to omega-3 fatty acids are known to promote many diseases, including cardiovascular, cancer, inflammatory, and autoimmune diseases, while increased amounts of omega-3 fatty acids (a lower ratio of omega-6 to omega-3 fatty acids) have an inhibitory effect. The ratio of omega-6 to omega-3 fatty acids in fresh pumpkin pulp is therefore favorable and is also supported by studies that recommend a balanced intake of omega fatty acids in the human diet [4].

Thus, considering the total amount of SFAs, MUFAs, and PUFAs, the content of palmitic, oleic, linoleic, and linolenic acids predominant among the evaluated fatty acids, and the determined ratio of omega-6 to omega-3, it can be concluded that fresh pumpkin pulp is a moderately lipophilic source compared to other pumpkin fractions, especially the seeds (Table 1), which contained the higher proportions of FAs. For example, with some exceptions, the palmitic acid content in fresh pulp is much lower than in other pumpkin fractions such as seeds, the peel, and flowers, ranging from 7.07 to 33.43%. In addition to palmitic acid, the content of oleic acid and linoleic acid is also higher in these fractions (with some exceptions), ranging from 0.72 to 45.63% and 34.77 to 67.24%, respectively, compared to the values determined in this work. On the other hand, fresh pulp contains a higher content of linolenic acid than other fractions (0.02 to 2.25%) (Table 1). Considering the nutritional properties of fresh pumpkin pulp [21] and the fatty acid content determined here, this food can therefore be considered an excellent choice for a balanced diet.

### 3.2. Fatty Acid Profile of Dried Pumpkin Pulp

Like fresh pumpkin pulp, the dried pulp also contained a high content of palmitic acid, oleic acid, linoleic acid, and linolenic acid, each as SFA, MUFA and PUFA. The palmitic acid content was between 10.72 and 23.02%, depending on the drying method (HAD, VAD, and CD) and the parameters used (Figure 1).

Compared to fresh pulp (11.20%), except for the 60 °C VAD sample (10.72%), drying significantly increased the palmitic acid content (*p* < 0.05); as the results showed (Figure 1a), all drying methods are suitable for further drying of pumpkin pulp. In particular, CD resulted in higher palmitic acid contents, which ranged from 17.32 to 23.02% depending on the temperature (50 to 70 °C). Although the values of palmitic acid in the HAD samples were within a narrow range of 12.44 to 17.88% regardless of the temperature (50, 60, and 70 °C) and airflow velocity (0.5, 1.0, and 1.5 m/s), the highest recoveries of this acid were found in the samples dried at a temperature of 60 °C (17.85%) and 50 °C (17.88%) with an airflow velocity of 0.5 m/s. VAD samples dried at 70 °C and 50 °C also gave high values for palmitic acid, i.e., 16.05 and 16.79%, respectively, but these were lower than the values found for CD and HAD pulp (50 and 60 °C, 0.5 m/s). With some exceptions, the mass fractions of other SFAs, such as stearic and behenic acid, are also higher in dried pulp than in fresh pulp, and they are significantly (*p* < 0.05) influenced by the type of drying process (Figure 1a). Stearic acid and behenic acid are between 1.53 and 5.42% and between 0.38 and 8.35%, respectively, depending on the drying process. Similar to palmitic acid, higher mass fractions of both acids are found in pulp dried under mild HAD conditions, e.g., at 50 or 60 °C and a low airflow velocity of 0.5 m/s. At these parameters, the following values were found: 3.00 and 5.21% for stearic acid and 4.66 and 6.11 % for behenic acid. It seems that the highest temperature of 70 °C has an influence on their decomposition regardless of the airflow velocity (0.5, 1.0 and 1.5 m/s). High values for stearic acid and behenic acid are also provided by VAD and CD of the pulp, with contents of 3.32 to 4.55% (VAD) and 1.81 to 5.42% (CD) for stearic acid and 5.94 to 8.34% (VAD) and 0.41 to 8.35% (CD) for behenic acid. Considering that palmitic acid, stearic acid, and behenic acid are the three main SFAs, their yield would influence the total amount of SFAs (Figure 1b). In addition, the total amount of SFAs would also be given at the optimum drying parameters determined for the individual SFAs. It can therefore be assumed that further drying of the SFAs can be carried out in the following order: CD at 70 °C (42.37%) > HAD at 60 °C and 0.5 m/s (35.20%) > VAD at 60 °C (33.84%) > VAD at 50 °C (32.58%) and HAD at 50 °C and 0.5 m/s (30.40%).

Among the MUFAs, oleic acid (C18:1n9) is the most abundant, with contents between 7.98 and 32.64%, depending on the used drying methods and conditions, which have a significant effect (*p* < 0.05) not only on its yield but also on the total amount of MUFAs (Figure 2).

The yield of oleic acid in the HAD pulp is highest at the lowest airflow velocity of 0.5 m/s, regardless of the drying temperatures of 50, 60, and 70 °C, i.e., 22.45, 24.15, and 21.33%, respectively. These values are close to those of fresh pulp (29.19%). Increasing the airflow velocity from 1.0 to 1.5 m/s in combination with a drying temperature of 50, 60, or 70 °C, the decomposition of the oleic acid takes place. The lowest values were obtained at 70 °C, i.e., 5.74 and 6.66% at 1.0 and 1.5 m/s, respectively. The CD pulp dried at 50 °C contains a similar amount of oleic acid (21.49%) as the HAD sample at a velocity of 0.5 m/s, while the temperature of 60 and 70 °C causes a degradation of oleic acid in CD. Compared to fresh or HAD and CD samples, the VAD pulp contains the higher mass fractions of oleic acid, namely 30.09 and 32.64% at 70 and 50 °C, respectively. On one hand, the HAD and CD processes are able to maintain the oleic acid content, i.e., the values are equal to those of the fresh pulp in some samples, which confirms the efficiency of drying when the drying parameters are chosen correctly. On the other hand, the VAD significantly increased the oleic acid content, so these results indicate that the use of vacuum and the absence of air prevents the oxidation of oleic acid, and, consequently, higher oleic acid values are obtained. Since the oleic acid content is the highest in VAD pulp dried at 50 °C, one would normally expect the total MUFA content to be highest in VAD pulp dried at this temperature. The results showed that the total amount of MUFA in the VAD samples dried at 50 °C was 33.61%, which would be a good method for further drying of pumpkin pulp due to the heat sensitivity of oleic acid. Comparing the amounts of oleic acid from fresh and dried pumpkin pulp with other unprocessed pumpkin fractions (seeds and peel), which range from 0.84 to 42.8% (Table 1), both appear to be a valuable source of oleic acid intake. 

Of the PUFAs, linoleic and linolenic acids are the most abundant in dried pumpkin pulp. The linoleic acid content ranges from 9.77 to 31.12% depending on the drying process, which has a significant (*p* < 0.05) influence on its yield (Figure 3). Of the drying methods used, CD drying at lower temperatures of 50 and 60 °C yielded the best linoleic acid contents of 31.12 and 30.73%, respectively. With a few exceptions, the HAD samples contained a higher linoleic acid content (16.81 to 29.06%, depending on temperature and airflow velocity) than fresh pulp (16.50%). The highest yield of 29.06 and 29.17% was obtained for samples dried at 50 °C and 1.0 m/s and 70 °C and 0.5 m/s, respectively. The VAD process yielded the lowest amounts of linolenic acid at 50 and 60 °C than fresh pulp, namely 13.55% and 9.77%. Compared to these results, the seed and peel fractions of other studies (Table 1) contained much higher values, ranging from 33.47 to 67.24%, depending on the pumpkin variety. However, when comparing the effect of the three drying methods used with the data from fresh pulp, it can be seen that, with some exceptions (Figure 3), drying significantly increases the linoleic acid yield, and if the pumpkin powder is to be used in the manufacture of pumpkin-based products, the dried form would be a valuable source of linoleic acid content instead of the fresh pulp. In addition to linoleic acid, the VAD samples also contained the lowest level of linolenic acid, ranging from 7.35 to 14.73%, compared to fresh pulp (Figure 3). The CD samples contained 12.36 to 20.63% linolenic acid and the HAD samples 9.73 to 41.23%, depending on the parameters used. The highest linolenic acid contents are obtained with HAD at the higher temperatures of 60 and 70 °C and a higher airflow velocity of 1.0 and 1.5 m/s (approx. 40%), which means that this drying technique is more suitable than CD and VAD. Obviously, the type of drying process had a significant effect on the linolenic acid yield, and in contrast to linoleic acid, the dried and fresh pumpkin samples analyzed in this work contained much higher levels of linolenic acid than the seeds and peels reported in other works (0.02 to 2.25%) (Table 1). This means that the linolenic acid contents determined here represent an important nutritional aspect, as their content together with linoleic acid influences the total content of omega-6 and omega-3 fatty acids (Figure 4). CD pulp had a higher total omega-6 fatty acid content (approx. 31%) than HAD and VAD pulp at 50 and 60 °C due to the high linoleic acid content. The total omega-3 fatty acids were mainly contained in the HAD pulp (approx. 41%), which is due to the high content of linolenic acid at higher temperatures of 60 and 70 °C and airflow velocities of 1.0 and 1.5 m/s. Thus, in accordance with the optimal drying process and the previously described parameters for the individual linoleic and linolenic acids, the total amounts of omega-6 and omega-3 fatty acids (Figure 4) are also given by CD and HAD. On the other hand, the different distribution of linoleic and linolenic acids in the dried samples affected the total amount of PUFAs (Figure 3). In this context, HAD seems to be the better drying method, i.e., the highest yields of 57.62, 58.39, and 59.26% are obtained for samples dried at 60 °C and 1.5 m/s and at 70 °C and 1.0 and 1.5 m/s, respectively, than for CD.

Overall, drying was found to significantly alter the fatty acid composition, with differences found between the drying methods used, i.e., HAD, VAD, and CD, and the specific conditions, e.g., temperature, airflow velocity, and pressure, as well as the drying duration [26]. These differences in fatty acid contents can be mainly attributed to the property of fatty acids to be susceptible to air oxidation and thermal decomposition. According to literature reports [58,59,60,61], lipid degradation involves chemical reactions such as oxidation, polymerization, and condensation, and the stability of fatty acids depends on various factors, including fatty acid composition, trace constituents, and environmental conditions. Oxidation is one of the most important reactions that fats undergo and is also accelerated by high temperatures [62,63]. Their combination leads to the formation of various compounds with a lower molecular weight, e.g., volatile compounds such as aldehydes, ketones, hydrocarbons, etc. Considering the above factors and the results of our study, we assume that all of the above factors influence the fatty acid composition and content. For example, the higher oxygen content in the air combined with the higher temperature led to a significant loss of some fatty acids. This was shown to be particularly true for saturated fatty acids such as palmitic, whose values were lower at higher airflow velocities of 1.0 and 1.5 m/s at 70 °C. As the sample is exposed to higher oxygen content and temperature during HAD and even to some extent during CD, oxidation of the oleic acid occurs, leading to its loss. Alternatively, the oxygen content can be minimized by VAD so that the oleic acid content is preserved. In contrast to oleic acid, linoleic and linolenic acids as PUFAs, which also have double bonds, are insensitive to decomposition by oxygen and heat, resulting in their highest contents in the CD or HAD samples. The matrix of the pumpkin pulp can also influence the stability of the fatty acids during drying. For example, if the fatty acids are combined with reactive components of the matrix at high temperatures, this can promote hydrolysis or secondary oxidation reactions, which in turn leads to their loss.

### 3.3. Fatty Acid Profile of Pumpkin By-Products

As already mentioned, the processing of pumpkin pulp for drying purposes produces large quantities of pumpkin by-products such as peel and seeds. Due to their rich nutritional and phytochemical composition, they could be used for therapeutic and pharmacological purposes that benefit human health [10,11,15,29,31,64,65].

**Figure 5:**
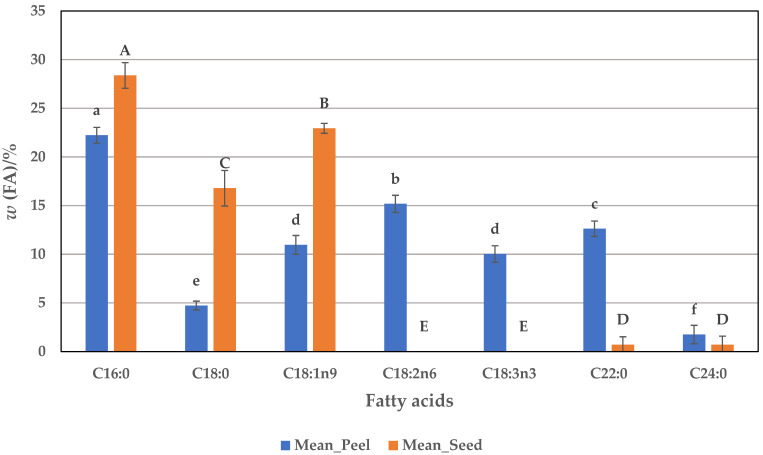
Profile of fatty acids (FAs) in pumpkin by-products (peel and seeds). Values are means ± standard deviations of three (*n* = 3) measurements. Different lowercase letters in the same column indicate significant differences (*p* < 0.05) between fatty acids (peel). Different uppercase letters in the same column indicate significant differences (*p* < 0.05) between fatty acids (seed).

In this part of the study, it was thus shown that the by-products obtained during the processing of pumpkin pulp can be effectively used for fatty acid extraction. In this context, the results (Figure 5) show that the peel and seeds contained 22.34 and 28.45% palmitic acid, respectively, which are significantly higher values than in fresh and dried pumpkin pulp. With a few exceptions, the stearic acid content in the peel (4.94%) is also higher than in the fresh and mostly dried pulp. However, with a value of 16.71%, the seeds are the largest source of stearic acid compared to the other samples examined. Not only this study, but also other investigations, have also shown a high stearic acid content in the seeds of 9.26 to 11.47% [54], 12.40% [37], and 14.27 to 14.43% [44]. The proportion of behenic acid is also high, especially in the peel, with a content of 12.99%, and tetracosanoic acid with values of 0.43 and 0.76 % for the seeds and peel, respectively. The distribution of SFAs in the peel and seeds is therefore more pronounced in both by-products than in fresh or dried pulp with values of 49.37 and 50.43%, respectively.

The seeds are an excellent source of oleic acid with a content of 22.36%, which is close to the values found for fresh (29.19%) and some dried samples (19.54–22.45%). The lower percentage of 9.85% is reported for the peel, so the total amount of MUFAs in the peel (11.13%) is also lower than in the seeds (23.98%) due to the lower proportion of oleic acid. Comparing the data of oleic acid from seeds and peels with other reported data, whose values range from 0.84 to 42.8% (Table 1), it seems that both by-products studied in this work can be considered a valuable source of oleic acid intake. Regarding linoleic and linolenic acids as the main PUFAs, the peel contains 14.49 and 10.98%, respectively, while these acids were not found in the seeds. In other studies, linoleic acid is reported in very high amounts of 34.77 to 67.24, depending on the variety (Table 1). However, in our work, we have not found such quantities of linolenic acid, which may be due to geographical origin or perhaps also to the atmospheric conditions during harvesting. Linoleic acid is not detected in the seeds, or only in very low amounts, of up to 2.25%, as shown in other studies (Table 1). In contrast, eicosapentaenoic acid (C20:5n3) is detected as a long-chain omega-3 acid with a content of 4.74% in the seeds, which contributes to the overall distribution of omega-3 acids and could be considered a useful food source. Eicosapentaenoic acid also contributes to the total PUFA content, as other acids could not be detected. In the peel, the total PUFA content is 29.13%, with the influence of linoleic and linolenic acids being more pronounced.

### 3.4. Fatty Acids Nutritional Quality Index

In this work, in addition to the already mentioned ratio of total omega-6 to total omega-3, the five other most commonly used nutritional indices for fatty acids, such as PUFA/SFA, PUFA/MUFA, index of atherogenicity (IA), index of thrombogenicity (IT), and index of hypocholesterolemia/hypercholesterolemia (HH), were evaluated in fresh and dried pumpkin pulp and by-products (Table 2).

The nutritional quality of the fatty acids and their positive or negative effects on the prevention of cardiovascular diseases are of great interest to the consumer. For example, the higher the PUFA/SFA index, the more positive the effect on cardiovascular health. According to Chen et al. [4], PUFA/SFA indices range widely, from 0.02 to 6.96, depending on the material studied, i.e., algae, plants, vegetable oil, shellfish, fish, meat, and dairy products. In this work, it is reported that the PUFA/SFA index for fresh pulp is 1.91, while for dried pulp it ranges from 0.75 to 3.33, depending on the drying method and parameters applied (Table 2). As shown, the PUFA/SFA index for VAD pulp is lower than for HAD and CD. The highest values are obtained for the samples with the codes 60, 1.5 m/s (2.98), 70 °C, 1.0 m/s (3.15), 70 °C, 1.5 m/s (3.30), and 50 °C, 1.0 m/s (3.33), practically, in the samples in which the higher amounts of linoleic and linolenic acid were determined. In contrast, the lowest ratio was found in the seeds and the peel with values of 0.09 and 0.59, respectively. Based on the fact that PUFAs can lower low-density lipoprotein cholesterol and serum cholesterol levels while SFAs contribute to high serum cholesterol levels, the data obtained here suggest that fresh and especially dried fruit pulp are suitable food sources compared to other foods studied [4].

In addition to PUFAs, MUFAs and their most abundant form, oleic acid, also reduced the risk of cardiovascular disease by lowering blood lipids, especially cholesterol. To illustrate the effect of MUFAs on health-related parameters, we also evaluated the MUFA/SFA indices (Table 2). The values obtained ranged from 0.32 to 1.12, depending on the drying process and parameters used. The lower values were mainly obtained for HAD samples with a high PUFA/SFA ratio, e.g., 70 °C, 1.0 m/s (0.35), 60, 1.5 m/s (0.39), and 70 °C, 1.5 m/s (0.41). In addition to the HAD pulp, the lower values of 0.32 were also determined for the CD samples dried at 70 °C. The higher MUFA/SFA ratio was mainly found for the HAD samples where higher oleic acid values were found, e.g., at 50, 60, and 70 °C, and an airflow velocity of 0.5 m/s, as well as for the CD samples dried at 50 °C (Table 2). The VAD samples also showed a high ratio due to the high proportion of oleic acid.

In addition to the ratios of PUFA/SFA and MUFA/SFA, the index of atherogenicity (IA) reported here represents the ratio between the sum of C12:0, C14:0, and C16:0 and the sum of unsaturated fatty acids. The SFAs mentioned, with the exception of the C18:0 acid, favor the adhesion of lipids to cells of the circulatory system and the immune system, while the unsaturated fatty acids reduce the content of phospholipids, cholesterols, and esterified fatty acids. The IA index ranges between 0.18 and 0.43 (Table 2), including fresh and dried products, confirming that drying processes have an influence on the modification of the SFA/UFA ratio. The values obtained are between those reported for various other foods such as seaweed, plants, vegetable oil, shellfish, fish, meat, and dairy products (0.03–5.13), suggesting that pumpkin is a beneficial nutrient source in both fresh and dried forms due to its lower IA index, i.e., that total cholesterol and low-density lipoprotein cholesterol levels are reduced. Although the IA values of the peel (1.23) and seeds (1.76) are lower than data reported for various other foods [1], both by-products could increase cholesterol levels compared to pulp. In addition to the IA index, the IT index can also be used to assess the potential impact of FA composition on cardiovascular disease. The IT index characterizes the thrombogenic potential of FAs, which indicates the tendency to form blood clots in the blood vessels, and provides the contribution of the different FAs, which indicates the ratio between pro-thrombogenic FAs (C12:0, C14:0, and C16:0) and anti-thrombogenic FAs (MUFAs and n-3 and n-6 families). The lower the IT value, the better the nutritional quality of the food and the more positive the effects on health. The IT values determined for dried pumpkin pulp are between 0.12 and 0.44, depending on the drying method and parameters used (Table 2). These low values indicate that both the dried and fresh pulp (IT = 0.17), as well as the peel (IT = 0.32) and seeds (IT = 0.24), are an excellent source of nutrients compared to other foods, whose values range between 0.16 and 5.04 [1].

According to Chen et al. [4], the last index evaluated in this paper, HH, reflects the effects of FA composition on cardiovascular disease more accurately than PUFA/SFA. The HH index characterizes the ratio between hypocholesterolemic fatty acids (C18:1) and PUFA and hypercholesterolemic FA (C12:0, C14:0, and C16:0). The HH ratio for dried pumpkin pulp is between 2.18 and 4.12, depending on the drying method and conditions. The lowest HH index is found for HAD with values of 2.9, 2.6, and 2.7 for samples dried at a temperature of 50, 60, and 70 °C with an airflow velocity of 1.5 m/s. In addition to this sample, low values were also found for the samples dried at 70 °C and 1.0 m/s and for the 70 °C, CD (2.18). The other dried samples and the fresh pulp (HH = 4.73) had a higher value, which means that there would be a more positive effect on cardiovascular health. On the other hand, the by-products had the lowest HH values, so it can be assumed that their impact on cardiovascular health is lower. In comparison, the other foods had HH values ranging from 0.32 to 15.0 [1]. Overall, the evaluated indices suggest that both the fresh and dried pumpkin pulp as well as the peel and seeds have a good nutritional fatty acid quality. With this fatty acid composition, the dried pulp in particular could influence the desired fatty acid composition and yield through the choice of drying parameters, leading to its function as an added benefit.

## 4. Conclusions

The fresh and dried pumpkin pulp contained a high content of palmitic acid, oleic acid, linoleic acid, and linolenic acid, each as SFA, MUFA, and PUFA. Drying had a significant effect on the composition and yield of the fatty acids studied, so the drying method used should be selected based on fatty acid preferences. As the results showed, CD yielded the higher amounts of SFAs and is therefore more suitable than HAD and VAD, although both gave good results depending on the parameters applied. On the other hand, VAD showed the highest amounts of oleic acid as MUFAs, so this method would be a good choice for further drying of pumpkin pulp due to the heat sensitivity of oleic acid. Regarding the drying methods used, CD at lower temperatures of 50 and 60 °C provided the best linoleic acid yield as PUFAs, while HAD at higher temperatures of 60 and 70 °C and airflow velocities of 1.5 and 1.0 m/s provided approximately 40% linoleic acid, indicating that this drying technique is more suitable than CD and VAD.

As drying processes have a significant impact on the yield of SFAs, MUFAs, and PUFAs and the final nutritional indices vary due to the different proportions of all fatty acids. Besides fresh and dried pumpkin pulp, the other two fractions, the peel and seeds, also contained the four mentioned fatty acids in representative amounts, indicating their valuable utilization after processing of the pulp.

## Figures and Tables

**Table 1:** Summary of previous fatty acid experiments carried out in pumpkin fractions (seeds, peel, and flower).

Cucurbita spp.	w (Fatty Acids)/%					
	Palmitic (16:0)	Stearic (18:0)	Oleic (18:1)	Linoleic (18:2)	Linolenic (18:3)	Reference
*C. maxima*	Seed					
	20.78–22.84	4.52–6.60	23.82–25.42	46.51–49.4	0.36–2.25	[10]
	17.41	6.56	18.12	56.98	0.28	[18]
	14.20	5.8	41.40	37.00	0.20	[36]
	17.90	12.40	18.80	50.70	/	[37]
	11.26	8.31	30.49	48.72	0.40	[38]
	15.65	7.80	29.30	45.90	/	[39]
	16.03–16.40	8.28–8.57	28.19–30.56	43.86–46.67	/	[40]
	17.58	7.62	25.54	47.45	0.69	[41]
	15.97	4.68	44.11	34.77	/	[42]
	Seed/Peel					
	11.55–12.06	7.39–8.31	31.98–32.87	43.73–44.84	0.39–0.43	[38]
	Flower					
	13.56–33.43	1.54–5.12	0.72–3.67	10.63–23.69	/	[43]
*C. moschata*	Seed					
	17.39	7.26	17.03	57.40	0.25	[18]
	20.88–21.03	14.27–14.43	15.92–16.41	39.75–40.73	2.12–2.13	[44]
	12.17–19.08	5.25–10.98	28.64–37.45	37.63–51.74	0.03–0.28	[45]
*C. pepo*	Seed					
	11.90–11.99	5.26–5.29	27.52–27.59	53.19–53.27	0.39–0.44	[16]
	15.83	7.33	23.86	52.11	0.12	[18]
	14–15.6	8.2–8.5	0.84–0.88	42–43.1	0.84–0.88	[46]
	11.43–11.56	4.94–5.21	24.63–25.21	56.90–57. 96	/	[47]
	14.21	7.29	33.39	45.10	/	[48]
	11.83–11.87	3.18–3.58	16.01–18.33	66.05–67.24	0.24–0.34	[49]
	10.4–10.5	6.4–7.3	37.7–38.3	44.3–44.9	/	[50]
	7.07–13.08	3.49–6.48	19.15–45.63	42.89–66.43	/	[51]
	12.5–14.0	4.6–5.03	24.6–28.1	52.8–55.8	0.3–1.0	[52]
*Non-defined cultivar*	Seed					
	11.05–11.21	7.81–8.05	31.22–32.92	45.66–48.81	0.14–1.48	[53]
	15.17–19.12	9.26–11.47	23.69–35.88	36.31–45.79	/	[54]
	Seed cake					
	11.5–11.6	6.0	42.6–42.8	38.3–38.4	0.2–0.4	[17]

**Table 2:** Fatty acids nutritional quality index of pumpkin pulp (fresh and dried) and by-products (peel and seeds).

Sample Code	PUFA/SFA	MUFA/SFA	Omega 6/Omega 3	IA	IT	HH
Fresh	1.91	1.71	0.94	0.20	0.17	4.73
50 °C, 1.5 m/s	1.72	0.43	0.57	0.41	0.21	2.90
50 °C, 1.0 m/s	3.33	0.70	1.16	0.22	0.15	3.06
50 °C, 0.5 m/s	1.17	0.77	1.12	0.36	0.30	3.45
60 °C, 1.5 m/s	2.98	0.39	0.45	0.18	0.13	2.60
60 °C, 1.0 m/s	1.11	0.80	1.90	0.39	0.30	3.64
60 °C, 0.5 m/s	0.75	0.71	1.48	0.42	0.44	3.43
70 °C, 1.5 m/s	3.30	0.41	0.50	0.19	0.12	2.68
70 °C, 1.0 m/s	3.15	0.35	0.41	0.25	0.12	2.57
70 °C, 0.5 m/s	2.74	1.12	1.18	0.30	0.18	3.54
50 °C, VAD	0.77	1.03	1.31	0.43	0.34	4.12
60 °C, VAD	0.76	0.56	2.29	0.21	0.34	3.69
70 °C, VAD	1.18	1.04	1.31	0.34	0.30	3.99
50 °C, CD	1.52	0.79	2.50	0.26	0.35	3.35
60 °C, CD	2.35	0.78	1.51	0.27	0.23	3.11
70 °C, CD	1.07	0.32	1.18	0.42	0.38	2.18
Peel	0.59	0.23	1.56	1.23	0.32	1.57
Seed	0.09	0.48	0.00	1.76	0.24	0.94

HAD—hot air drying (50, 60, and 70 °C, and 0.5, 1.0, and 1.5 m/s), VAD—vacuum drying, CD—conductive drying under atmospheric pressure.

## Data Availability

The original contributions presented in this study are included in the article. Further inquiries can be directed to the corresponding authors.
